# A survey on knowledge and attitudes towards molar-incisor hypomineralization among undergraduate and postgraduate students at the School of Stomatology, Wuhan University

**DOI:** 10.1186/s12903-022-02055-y

**Published:** 2022-01-26

**Authors:** Yanchen Liu, Miao He

**Affiliations:** 1 The State Key Laboratory Breeding Base of Basic Science of Stomatology (Hubei-MOST) & Key Laboratory of Oral Biomedicine Ministry of Education, School and Hospital of Stomatology, Wuhan University, 237 Luoyu Road, Wuhan, 430079 China; 2 Department of Paediatric Dentistry, School and Hospital of Stomatology, Wuhan University, Wuhan, 430079 China

**Keywords:** Clinical management, Dental developmental disease, Molar-incisor hypomineralization

## Abstract

**Background:**

Molar incisor hypomineralization (MIH) is a developmental dental disease, and its clinical management challenges dentists. This study aimed to investigate the knowledge about MIH and the attitudes towards learning more about MIH among undergraduate and postgraduate students attending the School of Stomatology, Wuhan University.

**Methods:**

This survey was based on a questionnaire modified based on previous studies. The questionnaire was sent to 540 undergraduate and postgraduate students from the School of Stomatology, Wuhan University. The questions covered their clinical experience, perceptions, clinical management, and preferences for further training. Data were analysed with the *Chi-square* test.

**Results:**

We collected 368 questionnaires (response rate: 68%). Among them, 89% (328/368) were eligible for analysis. Most respondents (80%) had heard of MIH, primarily from classroom teaching. However, only 40% of the students had observed the disease clinically, and a relatively low proportion of students were familiar with the aetiology, prevalence, differential diagnosis, and treatment of MIH. Most respondents were highly enthusiastic and had great expectations about further systematic teaching about MIH.

**Conclusion:**

Most students in this study had heard of MIH, but few were familiar with the principles of its differential diagnosis. Systematic teaching about MIH is warranted.

## Background

Molar-incisor mineralization (MIH) is a disease that causes hypomineralization of tooth enamel. It was first proposed by Weerheijm in 2001 [1], and it was officially defined as an independent disease by the European Academy of Paediatric Dentistry in 2003 [2]. The aetiology of MIH is currently believed to be related to genetic factors, environmental factors, drug factors, and general conditions that occur in utero and during the first three years after birth [3].

The global average prevalence of MIH is 14.2% [4], but it varies from country to country. The prevalence of MIH in children 6–12 years old in China ranges from 4.45 to 25.5% [5, 6]. The clinical manifestations of teeth mildly affected by MIH include clearly defined white and light-yellow opacities. In severe cases, the enamel may break down after eruption. There is no unified opinion on treatments for MIH, and currently, the treatments mainly aim to relieve symptoms. Previous studies have found that the oral health-related quality of life of children with MIH is lower than that of children without MIH [7]. The main reasons for the reduced quality of life include tooth sensitivity, which causes chewing discomfort, and coloured opacities on the front teeth, which causes aesthetic problems [8].

For dentists, it is important to distinguish MIH from other dental developmental defects. MIH has gradually received more attention over the years, and several surveys have been conducted on dentist awareness of MIH [9–11]. Dentists believe that MIH management presents quite a challenge. To date, only two surveys on student awareness have been conducted: one in Germany and one in Saudi Arabia [12, 13]. Given the high prevalence of MIH in China and the lack of awareness among dental students, the present study aimed to investigate the knowledge of MIH and the attitudes towards learning more about MIH among undergraduate and postgraduate students in the School of Stomatology, Wuhan University. We also evaluated whether knowledge of MIH was different among students at different academic levels. The results of this study might influence future curriculum planning.

## Methods

### Study design and procedures

This survey included undergraduates (fourth- and fifth-year undergraduates) and postgraduates (first-, second-, and third-year postgraduates) enrolled in the School of Stomatology, Wuhan University, in January 2021. All current students were included in the sample. Prior to participant enrolment, this study was approved by the Medical Ethics Committee of the School of Stomatology, Wuhan University (#HGGC-076).

Before the questionnaire was delivered, we conducted a presurvey to ensure that the questions were readily understood. For this study, the questionnaire was provided in electronic form and distributed in public discussion groups (WeChat and QQ software platforms, Tencent Corporate, Shenzhen, China) with assistance from Wenjuanxing (Questionnaire Collection Software, Ranxing Technology Corporate, Changsha, China). All questionnaires were completed voluntarily and anonymously. One week after the questionnaire was distributed, all of the participating groups were notified again as a reminder.

### Survey instrument

The questionnaire had three parts. The first part contained basic information, including each participant’s academic level. The second part included disease awareness; the source of knowledge acquisition; the degree of mastery over MIH aetiology, epidemiology, clinical manifestations, and differential diagnosis; the probability of clinical encounters; and the choice of treatment methods. The third part included the following three questions: Do you want to gain relevant knowledge about MIH? What would you most like to know? What are the principal ways to obtain professional dental knowledge? Between the first and second parts of the questionnaire, a brief introduction to MIH was provided to clarify the subject of the questionnaire and to avoid errors in understanding. The related pictures of MIH used in the questionnaire were from the treatment manual published by Ghanim et al. [14].

To avoid blank items in the questionnaire, the questionnaire was set to skip nonapplicable questions automatically. For example, when a respondent had not been involved in treating MIH, they were not asked about the preferred materials commonly used in clinical practice for treating MIH.

### Statistical analyses

We performed all statistical analyses with SPSS Ver. 26.0 (IBM, NY, USA). The descriptive statistical analyses are presented in distribution tables and frequency tables. The distributions were compared with the chi-square test. The reliability and validity of the questionnaire were verified with the Cronbach’s α index and Kaiser–Meyer–Olkin (KMO) value, respectively. The threshold for a significant difference was set to 0.05.

## Results

We collected 368 questionnaires from students at five different academic levels. The response rate of the questionnaire was 68% (Table 1), and the effective rate of the questionnaire was 89%. The reliability of the questionnaire was 0.852 (Cronbach's α index), and the validity of the questionnaire was 0.889 (KMO test).

**Table 1:** Recovery and valid questionnaires

	Recovery	Valid	Target number	RR (%)	ER (%)
G1	68	59	79	86	87
G2	69	64	79	87	93
G3	53	53	103	51	100
G4	90	82	135	67	91
G5	88	70	144	61	80
Total	368	328	540	68	89

*G1* 4th-year undergraduate, *G2* 5th-year undergraduate, *G3* 1st-year postgraduate

*G4* 2nd-year postgraduate, *G5* 3rd-year postgraduate

*RR* recovery rate, *ER* effective rate

Most students had heard of MIH (Table 2), and we found no significant difference between undergraduate and postgraduate students in this aspect (p > 0.05). Moreover, most students had heard about MIH in more than one context**.** As shown in Table 3, the most common source was classroom teaching, followed by clinical practice, lectures, journal articles, and other sources. Some students (39%) had observed MIH clinically. The proportions of students who had observed MIH clinically were significantly different among the different academic levels (p < 0.05). However, only 66 students (25%) were confident in identifying MIH, and the professed ability to identify MIH was significantly different among the different academic levels (p < 0.05). Few students knew the diagnostic principles of MIH. The proportions of students who knew the diagnostic principles of MIH were significantly different between academic levels (p < 0.05). All of the aetiologies of MIH (i.e., genetic factors, pregnancy and postnatal factors, drug factors, environment factors) were known by only 93 students (35%). Most students believed that the prevalence of MIH was less than 15%.

**Table 2:** Source of MIH knowledge and oral professional knowledge

Total	G1	G2	G3	G4	G5	Total
*Have you heard of MIH? (YES)*						
*Do you want to learn MIH more systematically? (YES)*						
Which part do you want to study in MIH?*						
Clinical manifestation	46 (58%)	47 (59%)	50 (49%)	72 (53%)	63 (44%)	278 (85%)
Differential diagnosis	43 (54%)	50 (63%)	48 (47%)	73 (54%)	61 (42%)	275 (84%)
Treatment methods	43 (54%)	50 (63%)	50 (49%)	78 (58%)	63 (44%)	284 (87%)
Aetiology	32 (41%)	38 (48%)	40 (39%)	68 (50%)	50 (35%)	228 (70%)
Epidemiology	22 (28%)	26 (33%)	30 (29%)	52 (39%)	37 (26%)	167 (51%)
Where do you usually learn oral knowledge?*						
Textbook	59 (75%)	52 (66%)	40 (39%)	59 (44%)	58 (40%)	268 (82%)
Journals	25 (32%)	33 (42%)	35 (34%)	62 (46%)	57 (40%)	212 (65%)
Internet	30 (38%)	37 (47%)	35 (34%)	63 (47%)	47 (33%)	212 (65%)
Lectures	9 (11%)	21 (27%)	29 (28%)	60 (44%)	50 (35%)	169 (52%)

*G1* 4th-year undergraduate, *G2* 5th-year undergraduate, *G3* 1st-year postgraduate, *G4* 2nd-year postgraduate, *G5* 3rd-year postgraduate

*These questions are multiple choice

**Table 3:** Students’ responses about the aetiology, diagnosis and prevalence of MIH

Total	47	47	47	69	54	264
Where did you learn about MIH?*						
Journals	2 (4%)	7 (15%)	5 (11%)	10 (14%)	13 (24%)	37 (14%)
Lectures	2 (4%)	5 (11%)	7 (15%)	21 (30%)	21 (39%)	56 (21%)
Classroom teaching	43 (91%)	38 (81%)	27 (57%)	48 (70%)	32 (59%)	188 (71%)
Clinical practice	3 (6%)	15 (32%)	25 (53%)	45 (65%)	23 (43%)	111 (42%)
*Have you met MIH in clinical? (YES)*	0	10 (21%)	26 (55%)	40 (58%)	28 (52%)	104 (39%)
What do you think is the aetiology of MIH?*						
Genetic factors	41 (87%)	41 (87%)	35 (74%)	56 (81%)	40 (74%)	213 (81%)
Pregnant and postnatal factors	41 (87%)	38 (81%)	40 (85%)	56 (81%)	42 (78%)	179 (68%)
Drug factors	29 (62%)	31 (66%)	19 (40%)	30 (43%)	31 (57%)	140 (53%)
Environment factors	28 (60%)	39 (83%)	24 (51%)	32 (46%)	38 (70%)	161 (61%)
All of above	22 (47%)	24 (51%)	11 (23%)	17 (25%)	19 (35%)	93 (35%)
*Do you know the principles of diagnosis of MIH? (YES)*	1 (2%)	3 (6%)	7 (15%)	22 (32%)	10 (19%)	43 (16%)
*What do you think the prevalence of MIH?*						
0–15%	28 (60%)	23 (49%)	25 (53%)	41 (59%)	39 (72%)	156 (59%)
15–30%	18 (38%)	22 (47%)	18 (38%)	27 (39%)	15 (28%)	100 (38%)
30–60%	1 (2%)	2 (4%)	4 (9%)	1 (1%)	0	8 (3%)
More than 60%	0	0	0	0	0	0 (0%)
*Have you did a treatment of MIH? (Yes)*	0	0	0	5 (7%)	4 (7%)	9 (3%)
*Which treatment would you provide for this is a newly erupted first molar with plaque on buccal surface?*						9 (3%)
Fluoride varnish	0	0	0	3 (4%)	4 (7%)	7 (3%)
Pit and fissure sealant	0	0	0	2 (3%)	0	2 (1%)
Observation	0	0	0	0	0	0
*Which treatment would you perfer to do for this newly erupted first permanent molar with post-eruptive breakdown?The tooth is sensitive to air and the patient is cooperative*						9 (3%)
Fluoride varnish	0	0	0	1 (1%)	1 (2%)	2 (1%)
Glass ionomer	0	0	0	1 (1%)	1 (2%)	2 (1%)
Composite resin	0	0	0	1 (1%)	0	1 (0%)
Occlusal veneer	0	0	0	2 (3%)	0	2 (1%)
Preformed crowns	0	0	0	0	2 (4%)	2 (1%)
Extract	0	0	0	0	0	0

*G1* 4th-year undergraduate, *G2* 5th-year undergraduate, *G3* 1st-year postgraduate, *G4* 2nd-year postgraduate, *G5* 3rd-year postgraduate

*These questions are multiple choice

Only 3% of respondents had performed an MIH treatment (Table 3). Among these students, most were more inclined to use a pit and fissure seal, instead of a fluoride varnish, for teeth mildly damaged by MIH. However, for teeth affected by MIH with moderate to severe dentin sensitivity but no pulp symptoms, no treatment was favoured significantly over the other choices.

Among the respondents who had observed MIH clinically, most (63%) were confident in distinguishing MIH from dental fluorosis, dentin hypoplasia, and other developmental dental diseases (Table 4). The rate of observing MIH clinically was 13%. The probability that students would encounter MIH clinically at frequencies of once per week, once per month, once per 6 months, once per year, and once per > 1 year were 13%, 34%, 38%, 7%, and 10%, respectively.

**Table 4:** Clinical experience of MIH

Total	0	10	26	40	28	104
*How often do you meet MIH in clinical?*						
Once a week	0	0	3 (12%)	8 (20%)	2 (7%)	13 (8%)
Once a month	0	4 (40%)	11 (42%)	11 (28%)	9 (32%)	35 (21%)
Once half a year	0	3 (30%)	8 (31%)	17 (43%)	11 (39%)	39 (23%)
Once a year	0	1 (10%)	2 (8%)	1 (3%)	3 (11%)	7 (4%)
Once more than a year	0	2 (20%)	2 (8%)	3 (8%)	3 (11%)	10 (6%)
*Can you distinguish MIH with other dental development disease? (Yes)*	0	3 (30%)	14 (54%)	30 (75%)	19 (68%)	66 (63%)

*G1* 4th-year undergraduate *G2* 5th-year undergraduate *G3* 1st-year postgraduate *G4* 2nd-year postgraduate *G5* 3rd-year postgraduate

The vast majority of students (90%) thought it was necessary to add MIH to the curriculum for future systematic teaching (Table 2). They felt that the most desirable aspects to learn were the clinical manifestations, differential diagnosis, and treatment methods. The most important ways to learn this knowledge were thought to be from textbooks, the literature, the internet, and lectures.

## Discussion

There are obvious differences in the MIH prevalence among previously published studies, possibly due to differences in the populations, research methods, diagnostic criteria, etc. Compared to unaffected children, children with MIH had higher risks of caries and required more dental treatments [15]. Severe MIH can affect both the quality of life and oral function in children [16]. Without intervention, after two years, molars affected by mild MIH progress to moderate or severe MIH, with enamel breakdown [17]. If we do not initiate preventive measures, the cost of treating MIH will become a heavy burden on the country and the individuals [18]. Therefore, it is important to study the aetiology, treatment, and awareness of MIH.

Judging from the current data, the situation is not optimistic. Although 80% of students had heard of MIH, only 20% of the respondents thought they could accurately evaluate it. Student enthusiasm for learning MIH-related knowledge was very high, which showed that we need to increase the teaching of MIH-related theoretical knowledge.

For the question of "How often do you encounter MIH clinically?", the results showed a far lower frequency than the frequency experienced by the authors in encountering patients with MIH in clinical settings. This discrepancy might be explained by several reasons. First, the authors work in the paediatric dentistry department; thus, we have come into contact with a large number of patients with MIH. Second, the authors have a better understanding of the theoretical knowledge of MIH. In contrast, the respondents were studying different majors and lacked sufficient opportunities to encounter patients with MIH clinically or to study MIH systematically.

We found no difference among the different academic levels in regards to wishing to learn more about MIH. Most students desired to learn the clinical manifestations, differential diagnosis, and treatment for MIH. This result reflected the urgent clinical need for knowledge about the principles of MIH diagnosis and treatment.

The different academic levels acquired knowledge through different sources. As the respondents aged, they gradually changed their ways of acquiring knowledge, from textbooks to journals, lectures, and then social platforms (e.g., blogs). On the one hand, this trend reflected the convenience of current networks; on the other hand, it also reflected the dentist’s growth process, from passive learning to active learning. By searching for reading materials, students can improve their knowledge reserve and build their own experience system.

In this survey, nearly 40% of interviewees had observed MIH clinically, but only 10% had experienced treating MIH. At present, there is no global consensus on MIH treatment methods, and treatment principles often lack clear indicators. In 2017, Steffen et al. proposed an MIH treatment-need index (MIH-TNI) with an MIH treatment-need coefficient, which could assist in establishing an MIH treatment system [19].

Compared with the data in the two published studies thus far, the awareness of Chinese students is lower than that of students in Germany and Saudi Arabia [12, 13]. However, Chinese students’ ability to diagnose MIH increases with grades, which may be due to their increase in clinical experience. In addition, we also compared the awareness between students in our study with oral health care practitioners (OHCPs) who have been practising for many years, including general dental practitioners (GDPs) and oral health therapists (OHTs) [10]. As many as 70% of OHCPs recognise MIH, which is much higher than that of the dental students [13, 20]. In the future, accordingly, the introduction of MIH in paediatric dentistry courses at the undergraduate and graduate levels is necessary, and surveys should be conducted before and after teaching and learning to evaluate the outcomes.

## Conclusion

The present study showed that students attending the School of Stomatology, Wuhan University had some understanding of MIH, but their knowledge was not comprehensive. They had a weak grasp of the key diagnostic and treatment aspects, and feel a strong need to learn more about MIH. Therefore, based on our research results, we recommend that comprehensive, systematic training on MIH should be required at the undergraduate level, and additional training should be offered at the postgraduate level.

## Data Availability

The dataset used and/or analysed during the current study is available from the corresponding author on reasonable request.
